# The effect of chronic exposure to high palmitic acid concentrations on the aerobic metabolism of human endothelial EA.hy926 cells

**DOI:** 10.1007/s00424-016-1856-z

**Published:** 2016-07-14

**Authors:** Izabela Broniarek, Agnieszka Koziel, Wieslawa Jarmuszkiewicz

**Affiliations:** Department of Bioenergetics, Adam Mickiewicz University, Umultowska 89, 61-614 Poznan, Poland

**Keywords:** Mitochondria, Free fatty acids, Endothelial cells, Oxidative metabolism, Mitochondrial respiration

## Abstract

A chronic elevation of circulating free fatty acids (FFAs) is associated with diseases like obesity or diabetes and can lead to lipotoxicity. The goals of this study were to assess the influence of chronic exposure to high palmitic acid (PAL) levels on mitochondrial respiratory functions in endothelial cells and isolated mitochondria. Human umbilical vein endothelial cells (EA.hy926 line) were grown for 6 days in a medium containing either 100 or 150 μM PAL. Growth at high PAL concentrations induced a considerable increase in fatty acid-supplied respiration and a reduction of mitochondrial respiration during carbohydrate and glutamine oxidation. High PAL levels elevated intracellular and mitochondrial superoxide generation; increased inflammation marker, acyl-coenzyme A (CoA) dehydrogenase, uncoupling protein 2 (UCP2), and superoxide dismutase 2 expression; and decreased hexokinase I and pyruvate dehydrogenase expression. No change in aerobic respiration capacity was observed, while fermentation was decreased. In mitochondria isolated from high PAL-treated cells, an increase in the oxidation of palmitoylcarnitine, a decrease in the oxidation of pyruvate, and an increase in UCP2 activity were observed. Our results demonstrate that exposure to high PAL levels induces a shift in endothelial aerobic metabolism toward the oxidation of fatty acids. Increased levels of PAL caused impairment and uncoupling of the mitochondrial oxidative phosphorylation system. Our data indicate that FFAs significantly affect endothelial oxidative metabolism, reactive oxygen species (ROS) formation, and cell viability and, thus, might contribute to endothelial and vascular dysfunction.

## Introduction

Lining the inner lumen of blood vessels, the endothelium constitutes the interface between blood and the vessel wall, which is important in vascular homeostasis. Endothelial cells play a key role in sensing and responding to various physiological blood components, but they are also challenged by pathological stimuli, which may exert harmful effects on the vascular system via the excessive formation of reactive oxygen species (ROS). Endothelial cells are the first target for elevated levels of lipid-derived free fatty acids (FFAs) in the plasma, one of multiple risk factors of endothelial dysfunction [16, 24]. Abnormally enhanced levels of circulating FFAs are observed in type 2 diabetes and obesity that, together with insulin resistance and inflammation, facilitates fat accumulation [1]. Insulin resistance and the local biosynthesis of tumor necrosis factor alpha (TNFα) promote lipolysis in the adipose tissue, which stimulates the release of an additional pool of FFAs that may become preferential substrates for mitochondrial oxidation. Thus, metabolic diseases can cause a mismatch between lipid supply and uptake in adipose tissue, leading to increasing levels of FFAs, triglycerides, and cholesterol in the blood plasma [10]. Because endothelial cells are not able to store large amounts of lipids, pathologically elevated blood lipid levels are a dangerous metabolic challenge for endothelial mitochondria, which threatens their function and integrity. Because damage to endothelial cells is an important event in the pathogenesis of atherosclerosis and vascular disease [16, 24], the apoptotic effects of FFAs on human endothelial cells could result in serious health consequences [19]. Moreover, FFAs are important modulators of the cellular production of ROS [21]. They can cause oxidative stress and alternations in mitochondrial structure and function [20].

Emerging experimental evidence suggests an important role for endothelial mitochondria in the development of many cardiovascular diseases [5, 23, 25, 27]. Endothelial mitochondria may function as sensors of changes in the local environment (in particular, changes in the composition of the perfusate), contributing to the survival of endothelial cells under oxidative stress. On exposure to cardiovascular risk factors, endothelial mitochondria produce excessive ROS, which are important signaling molecules in vascular endothelial cells [29].

Fatty acids are important sources of energy because, when metabolized, they yield large quantities of ATP. Endothelial cells mainly use anaerobically metabolized glucose to generate ATP [9]. However, primarily glycolytic endothelial cells possess highly active mitochondria, with a functioning energy-dissipating system (uncoupling protein 2, UCP2) [14]. UCP2 is a regulator of mitochondrial ROS generation and can antagonize oxidative stress-induced modifications of endothelial mitochondrial function [23]. It has been shown that high glucose exposure induces a shift in endothelial aerobic metabolism toward the oxidation of lipid-derived FFAs [14].

Many questions must be addressed with respect to understanding the physiological role that mitochondria plays in endothelial cells and the contribution of endothelial mitochondria to vascular function and disease. Hyperlipidemia represents a major risk factor for the development of vascular dysfunction and atherosclerosis. The unfortunate contribution of triglyceride-derived FFAs to vascular disorders is not completely understood. Thus, the aim of the present study was to elucidate the effects of a chronic 6-day exposure to high palmitic acid (PAL) concentrations on cultured human endothelial EA.hy926 cells. Cell viability and proliferation and superoxide formation and mitochondrial respiratory function, including the respiratory response to different reducing fuels and mitochondrial oxidative capacity, were monitored in cells treated with 100 μM or 150 μM PAL. Moreover, we examined the effect of the chronic exposure of growing endothelial cells to high PAL concentrations on mitochondria by measuring their respiratory activities in phosphorylating and non-phosphorylating states, the yield of ATP synthesis, mitochondrial membrane potential (mΔΨ), and mitochondrial protein-mediated uncoupling.

## Material and methods

### Cell culture and cell fraction preparation

**Fig. 1:**
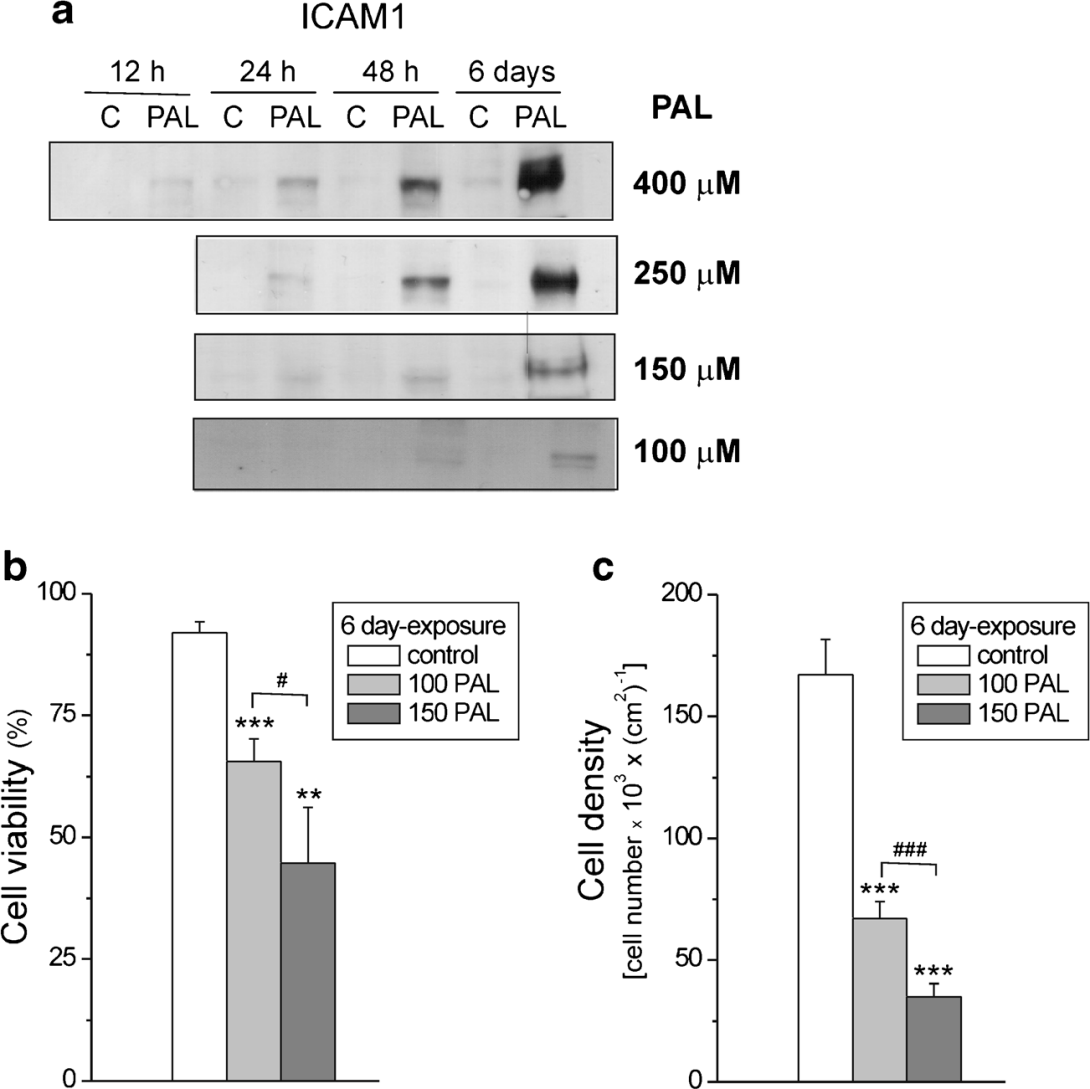
PAL-induced impairment of endothelial EA.hy926 cells. **a** Dose- and time-dependent levels of ICAM1 protein expression in control (*C*) and PAL-treated (*PAL*) endothelial EA.hy926 cells. PAL-treated cells were cultured for 12, 24, and 48 h or for 6 days with 100–400 μM PAL. Examples of immunoblots are shown. The same amount of proteins (40 μg) were loaded into each lane. Viability (**b**) and density (**c**) of cells in culture dishes after chronic exposure (the 6-day exposure) to 100 μM PAL (100 PAL) or 150 μM PAL (150 PAL). **b, c** Results are presented as the means ± SD; *n* = 7. *p* < 0.05 (#), *p* < 0.01 (**), or *p* < 0.001 (*** or ###). *Asterisks* (**, ***) are comparison vs. control values. *Number signs* (#, ###) are comparison between values obtained for cells grown in the presence of 100 μM PAL and those grown in the presence of 150 μM PAL

We used the stable human endothelial cell line EA.hy926 (ATCC® CRL-2922™), which was originally derived from a human umbilical vein [7]. Cells were grown in Dulbecco’s modified Eagle medium (DMEM) supplemented with 10 % fetal bovine serum (FBS), 1 % l-glutamine, 2 % hypoxanthine-aminopterin-thymidine (HAT), and 1 % penicillin/streptomycin in a humidified 5 % CO_2_ atmosphere at 37 °C. Except for the determinations of the time course and dose-dependency of the intercellular adhesion molecule 1 (ICAM1) expression level (Fig. 1), the EA.hy926 cells were cultured for 6 days in DMEM culture in the absence (FFA-free control conditions) or presence of 100 or 150 μM PAL (representing high FFA conditions). In the FFA-treated cells, albumin-conjugated FFAs (i.e., 100 or 150 μM PAL at ratios of fatty acid to albumin of 2.4:1 and 3.6:1, respectively) were added to the culture medium at the inoculation step. PAL makes up a major fraction of the saturated FFAs in the plasma. Cell culture media were changed every 3 days throughout the culturing period. The EA.hy926 cells were cultured in 140-mm dishes until they reached approximately 90–100 % confluence. Cells that were used in this study were between passages 5 and 12.

EA.hy926 cell cultures from both the control and high PAL cultures were harvested with trypsin/EDTA, rinsed twice with phosphate-buffered saline (PBS) (containing 10 and 5 % FBS, respectively), and centrifuged at 1200×*g* for 10 min. Subsequently, the cells were washed in cold PBS medium and then centrifuged again. The final cell pellet was resuspended in the PBS medium (1 g of cells per 2 ml of medium) and kept on ice. Protein content was determined using the Bradford method (Bio-Rad). The yield of the harvested cells differed significantly between the control and the 100 and 150 μM PAL-treated cells. Namely, 4.3 ± 0.4, 2.5 ± 0.17, and 0.6 ± 0.05 g of cells (SD, *n* = 12), respectively, were harvested from 50 dishes of each culture (when all types of cells were inoculated at the same density).

### Measurements of cell respiration

The detached untreated and PAL-treated (100 or 150 μM PAL) EA.hy926 cells were resuspended in cold DMEJ ion medium (modified DMEM medium), instead of PBS medium, containing 5.4 mM KCl, 0.8 mM MgSO_4_, 110 mM NaCl, 44 mM NaHCO_3_, 1.1 mM NaH_2_PO_4_, and 10 mM Na/Na buffer (pH 7.5). Cellular oxygen uptake was measured at 37 °C using a Clark-type electrode (Hansatech) in 0.7 ml of DMEJ medium.

The mitochondrial function in detached EA.hy926 cells was determined polarographically as previously described [14]. The following respiratory substrates were used: 5 mM pyruvate, 5.5 mM glucose, 3 mM glutamine, or 0.3 mM PAL. To estimate the contribution of ATP-linked oxygen consumption rate (OCR) and non-ATP-linked OCR (proton leak) to the basal respiratory rate, oligomycin (1 μg/ml) was added to inhibit ATP synthesis. Subsequently, the proton ionophore (uncoupler) carbonyl cyanide 4-(trifluoromethoxy) phenylhydrazone (FCCP, 0.5 μM) was added to determine the maximal oxygen uptake that the cells could sustain. Finally, cyanide (0.5 mM) was added to inhibit Complex IV (cytochrome *c* oxidase, COX) and thereby block the entire mitochondrial cytochrome pathway. In the presence of cyanide, no residual (non-mitochondrial) respiration was observed.

### Mitochondrial isolation and cytosolic fraction preparation

Mitochondria were isolated from EA.hy926 cells using a very efficient isolation procedure that produces highly active and well-coupled mitochondria [14]. After the cells were harvested and washed in PBS, cells were resuspended in PREPI medium (0.25 M sucrose, 1.5 mM EDTA, 1.5 mM EGTA, 0.2 % BSA, and 15 mM Tris/HCl, (pH 7.2)) at a ratio of 3 ml of medium per 1 g of cells. The cells were then homogenized via ten passes with a tight Dounce homogenizer, and the homogenates were subsequently centrifuged at 1200×*g* for 10 min. The pellets were resuspended, and the cells were once again homogenized (10–8 passes) and centrifuged to collect the mitochondria remaining in the pellet. The supernatants were combined and then centrifuged at 1200×*g* for 10 min, and the resulting supernatants were then centrifuged at 12,000×*g* for 10 min. The mitochondrial pellets were washed with a PREPII medium containing 0.25 M sucrose and 15 mM Tris/HCl (pH 7.2) and centrifuged at 12,000×*g* for 10 min. All of the steps were performed at 4 °C. The final mitochondrial pellet was resuspended in the PREPII medium. The yields of the isolated mitochondria were equal to 3.3 ± 0.6, 2.8 ± 0.5, and 1.33 ± 0.3 mg of mitochondrial protein per gram of cells (SD, *n* = 10) for cells grown under control conditions and in the presence of 100 or 150 μM PAL, respectively.

To obtain cytosolic fractions for enzymatic measurements, cells were homogenized in one step in the PREPII medium with the Dounce homogenizer (30 passes). The homogenates were subsequently centrifuged at 1200×*g* for 10 min. After spinning down the unbroken cells and cell debris, the supernatants were collected for measurements of citrate synthase (CS) activity and COX activity.

### Measurements of mitochondrial respiration and membrane potential

Mitochondrial respiration and membrane potential (mΔΨ) were measured in isolated endothelial mitochondria as previously described [14]. Oxygen uptake was determined polarographically using a Rank Bros. (Cambridge UK) oxygen electrode or a Hansatech oxygen electrode in either 1.4 or 2.8 ml of standard incubation medium (at 37 °C), which consisted of 150 mM sucrose, 2.5 mM KH_2_PO_4_, 2 mM MgCl_2_, 20 mM Tris/HCl (pH 7.2), and ±0.1 % BSA, with either 0.7 or 2 mg of mitochondrial protein. O_2_ uptake values are presented in nmol O_2_ min^−1^ mg^−1^ protein. Membrane potential was measured simultaneously with oxygen uptake using a tetraphenylphosphonium (TPP^+^)-specific electrode. The TPP^+^-electrode was calibrated based on four sequential additions (0.4, 0.4, 0.8, and 1.6 μM) of TPP^+^. After each run, 0.5 μM FCCP was added to release the TPP^+^ for a baseline correction. To calculate the mΔΨ value, the matrix volume of endothelial mitochondria was assumed to be 2.0 μl mg^−1^ protein. The calculation assumed that the TPP^+^ distribution between the mitochondria and the medium followed the Nernst equation. The mΔΨ values were corrected for TPP^+^ binding using the apparent external and internal partition coefficients of TPP^+^ [28]. This correction decreased the calculated mΔΨ values (approx. 30-mV shift), but it did not influence the changes in the resulting membrane potential (relative changes). The values for mΔΨ are given in millivolts.

Phosphorylating respiration was measured using 150 μM ADP (pulse), and uncoupled respiration was measured using up to 0.5 μM FCCP. Only high-quality mitochondria preparations, i.e., with an ADP/O value of approximately 2.2 and a respiratory control ratio of approximately 2.6–3.2 (with malate as a respiratory substrate), were used in the experiments. Non-phosphorylating (resting state) respiration measurements were performed in the absence of exogenous ADP.

The proton leak measurements were performed with 10 mM succinate (plus 2 μM rotenone) as an oxidizable substrate, in the presence of 1.8 μM carboxyatractyloside and 0.5 μg/ml oligomycin, which inhibit the activities of the ATP/ADP antiporter and ATP synthase, respectively. The response of proton conductance to its driving force can be expressed as the relationship between the oxygen consumption rate and the mΔΨ (flux-force relationship) when varying the potential via titration with respiratory chain inhibitors. To decrease the rate of the coenzyme Q-reducing pathway, succinate dehydrogenase was titrated with malonate (up to 4 mM). To induce UCP2 activity, 14 μM PAL was used. To inhibit UCP2 activity, 4 mM guanosine triphosphate (GTP) was applied.

### Measurement of enzyme activities

The activity of CS was determined by spectrophotometrically tracking the formation of DTNB-CoA at 412 nm using a UV 1620 Shimadzu spectrophotometer as described previously [14, 32]. The reaction mixture contained 100 mM Tris/HCl (pH 8.0) 100 μM acetyl CoA, 100 μM 5,5′-di-thiobis-(2-nitrobenzoic acid) (TNB), 0.1 % Triton X-100, and 100 μM oxaloacetate. The activity of lactate dehydrogenase (LDH) was measured spectrophotometrically at 340 nm by following the oxidation of NADH (150 μM) mixed with pyruvate (10 mM) in 50 mM Tris/HCl (pH 7.3) [15]. The activity of both enzymes was measured in 50 μg of protein from the cytosolic fractions obtained from living endothelial cells.

The maximal activity of COX and the integrity of the outer mitochondrial membrane were assessed polarographically as described previously [14]. The maximal activity of COX was assessed in 2 mg of cellular protein or in 0.25 mg of mitochondrial protein without exogenously added respiratory substrate and in the presence of sequentially added antimycin A (10 μM), 8 mM ascorbate, 0.06 % cytochrome *c*, and up to 2 mM N,N,N′N′-tetramethyl-*p*-phenylenediamine (TMPD). The rate of oxygen consumption following the addition of TMPD reflects the maximal O_2_ consumption by COX (Complex IV). The integrity of the outer mitochondrial membrane was assayed in mitochondrial fractions as the latency of COX activity during the oxygen uptake measurements in the absence and presence of exogenous cytochrome *c.*

All enzymatic measurements were performed at 37 °C with continuous stirring.

### Determination of superoxide anion formation in cells

ROS production was detected using a nitroblue tetrazolium (NBT) assay with untreated and PAL-treated EA.hy926 cells. NBT (yellow water soluble) was reduced by superoxide to formazan-NBT (dark-blue water insoluble). The assay was performed by incubating detached cells (0.2 mg of protein in a 1-ml DMEM) or adherent cells (grown in dishes) with 0.2 % NBT under agitation. The samples were incubated for 1 h (37 °C) in the presence or absence of 10 μM diphenyleneiodonium (DPI) (an inhibitor of NADPH oxidase and endothelial nitric oxide synthase (eNOS), enzymes involved in endothelial ROS formation), and the adherent cells were harvested. The cells were centrifuged (1200×*g* for 10 min at 4 °C), the supernatant was removed, and the formazan-NBT was dissolved in 200 μl of 50 % acetic acid by sonication (three pulses of 10 s each; Bandelin Electronic). The samples were briefly centrifuged (spun down), and the absorbance of the supernatant was determined at 560 nm using a UV 1620 Shimadzu spectrophotometer.

Additionally, mitochondrial superoxide formation was measured using MitoSox Red (Invitrogen), a specific fluorescent mitochondrial superoxide indicator. The assay was performed by incubating detached cells (50 μg of protein in 1 ml medium) or adherent cells (grown in 96-well plates) with 5 μM MitoSox in PBS containing 5.5 mM d-glucose and 5 mM pyruvate for 10 min at 37 °C. Cells were washed twice with PBS. Fluorescence emission at 595 nm under 510-nm excitation was recorded using an Infinite M200 PRO Tecan multimode reader.

### Measurement of mΔΨ in cells

To measure mΔΨ of the control and PAL-treated EA.hy926 cells, JC-1 (Invitrogen), a mΔΨ indicator in living cells, was used. The assay was performed by incubating detached cells (50 μg of protein in 1-ml medium) with 2 μM JC-1 in PBS containing 5.5 mM d-glucose and 5 mM pyruvate for 10 min at 37 °C. After incubation, cells were washed five times with PBS. Red aggregate fluorescence emission at 590 nm under 535-nm excitation was recorded using an Infinite M200 PRO Tecan multimode reader in 96-well plates (5 μg of cell protein per well). Measurements were performed in the absence and presence of 0.04 μM FCCP (a protonophore) to confirm that directional changes in fluorescence can be attributed to mitochondrial mΔΨ. For control and PAL-treated cells, FCCP-sensitive changes in fluorescence were calculated.

### Trypan blue cell viability assay

Both living and dead cells were harvested from cultures. After cell harvesting, 0.4 % trypan blue solution was added (1:1 *v*/*v*), and cell viability was determined using a Countess Automated Cell Counter (Invitrogen). In a trypan blue exclusion assay, cells that take up the dye are either necrotic or apoptotic.

### Determination of protein levels via immunoblotting

RIPA buffer (150 mM NaCl, 1 % Triton X-100, 0.5 % Na deoxycholate, 0.1 % SDS, 50 mM Tris (pH 8.0)) was used to lyse endothelial cells. The cellular and mitochondrial fractions were isolated in the presence of protease inhibitors (Sigma). The proteins were separated on an 8–12 % SDS-PAGE gel. The Spectra™ Multicolor Broad Range Protein Ladder (Fermentas) was used as a molecular weight marker. The following primary antibodies were used: rabbit monoclonal anti-intercellular adhesion molecule 1 (ICAM1, 89 kDa) (an53013, Abcam), mouse monoclonal anti-β actin (42 kDa) (CP01, Calbiochem), mouse monoclonal anti-hexokinase I (HKI, 120 kDa) (sc-80978, Santa Cruz Biotechnology), rabbit monoclonal anti-acyl-coenzyme A dehydrogenase (ACADS, 44 kDa) (ab-154823, Abcam), mouse monoclonal lactate dehydrogenase (LDH, 35 kDa) (sc-133123, Santa Cruz Biotechnology), rabbit polyclonal glutamate dehydrogenase (GDh, 61 kDa) (ab89967, Abcam), purified goat polyclonal anti-E3-binding protein of pyruvate dehydrogenase (E3BP, 54 kDa) (sc-79236, Santa Cruz Biotechnology), rabbit polyclonal anti-UCP2 (33 kDa) (ab97931, Abcam), rabbit polyclonal anti-UCP3 (34 kDa) (ab3477, Abcam), rabbit monoclonal antimitochondrial superoxide dismutase 2 (SOD2, 25 kDa) (ADI-SOD-110, Enzo Life Sciences), rabbit polyclonal anticitrate synthase (CS, 52 kDa) (ab-96600, Abcam), mouse monoclonal antimitochondrial marker (MTC02, 60 kDa), and the MitoProfile® total OXPHOS human antibody cocktail (MS601, MitoScience) containing antibodies raised against subunits of Complex I (20 kDa subunit NDUFB8), Complex II (30 kDa subunit), Complex III (subunit Core 2, 47 kDa), Complex IV (COXII, 24 kDa), and ATP synthase (subunit α, 57 kDa). Appropriate horseradish peroxidase-conjugated secondary antibodies were used. The expression levels of COXII or a mitochondrial marker (for the mitochondrial fractions) and of β-actin (for the cell fractions) were used as loading controls for normalization. Protein bands were visualized using the Amersham ECL system and digitally quantified using the GeneTools 4.03 software package.

### Statistical analysis

Data are presented as the means ± SD obtained from at least five to ten independent experiments (cell suspension preparations or mitochondrial isolations), and each determination was performed at least in duplicate in this study. Significant differences were determined via unpaired *t* tests or ANOVAs (followed by Tukey’s post hoc comparisons for *P* < 0.05 from an ANOVA). Differences were considered to be statistically significant at *p* < 0.05 (* or #), *p* < 0.01 (** or ##), or *p* < 0.001 (*** or ###). Asterisks (*, **, ***) represent comparisons of data obtained between PAL-treated cells and untreated control cells. Number signs (#, ##, ###) represent comparisons between PAL-treated cells, i.e., cells grown in the presence of 100 μM PAL compared to cells grown in the presence of 150 μM PAL.

## Results

### Time course and dose response of PAL-induced inflammatory marker and cytotoxicity in cultured endothelial EA.hy926 cells

**Fig. 2:**
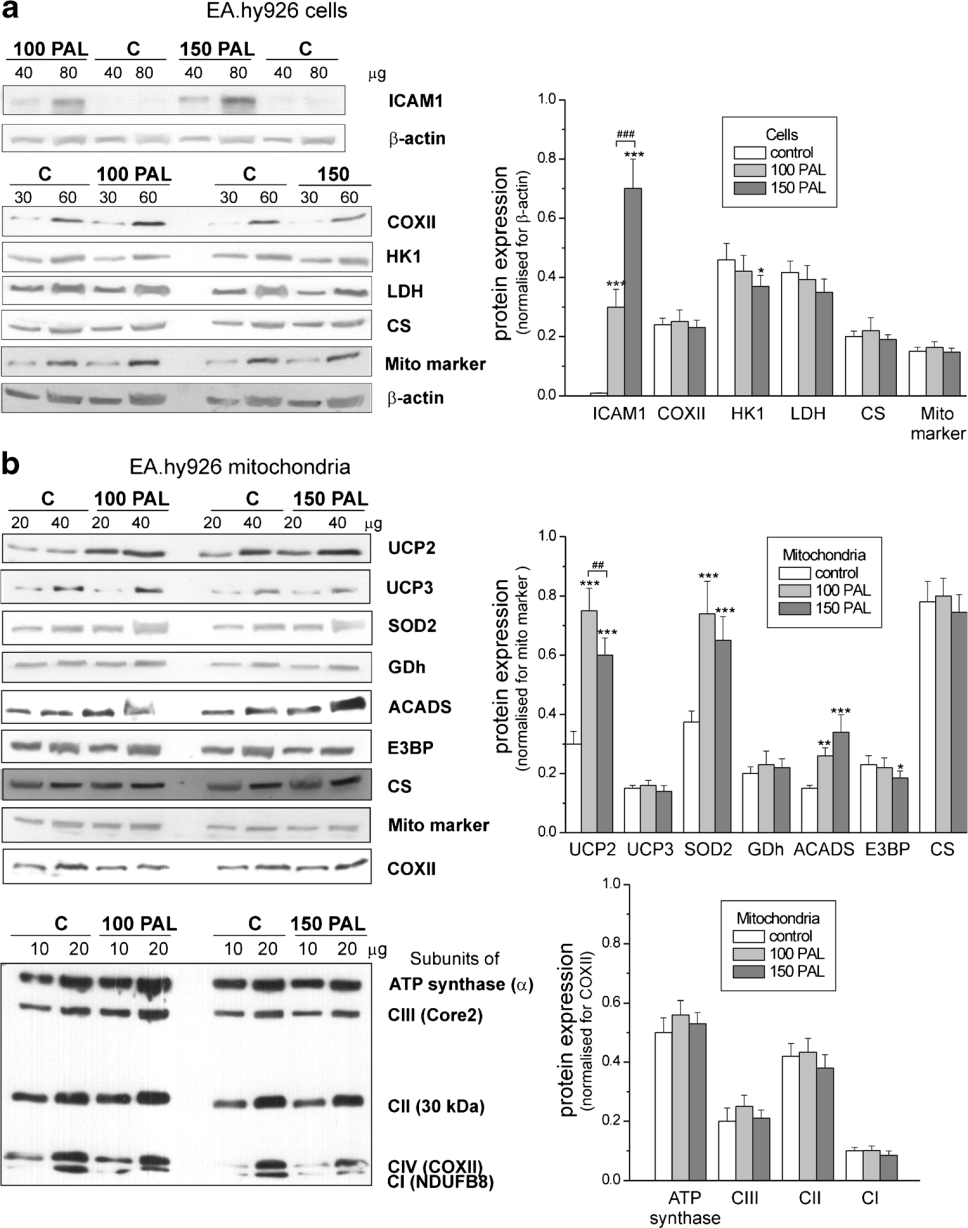
Determination of protein levels in endothelial cells grown for 6 days in the presence of 100 μM PAL (100 PAL), 150 μM PAL (150 PAL), or under control (C) conditions (**a**) and in mitochondria isolated from these cells (**b**). **a** Representative Western blots and analyses of the protein expression of ICAM1, cytochrome *c* oxidase (*COXII*), lactate dehydrogenase (*LDH*), hexokinase I (HKI), citrate synthase (*CS*), and mitochondrial marker (*Mito marker*). **b** Representative Western blots and analyses of the protein expression of the uncoupling proteins UCP2 and UCP3, superoxide dismutase (*SOD2*), glutamate dehydrogenase (*GDh*), acyl-coenzyme A dehydrogenase (*ACADS*), pyruvate dehydrogenase (*E3BP*), and citrate synthase (*CS*) (*upper panel*) and particular subunits of ATP synthase, Complex III (*CIII*), Complex II (*CII*), Complex IV (*CIV*, *COXII*), and Complex I (*CI*). Expression levels normalized for β actin (**a**), Mito marker (**b** *, upper panel*) or COXII (**b** *, lower panel*) protein abundance is shown. Results are presented as the means ± SD; *n* = 8. *p* < 0.05 (*), *p* < 0.01 (** or ##), or *p* < 0.001 (*** or ###). *Asterisks* (*, **, ***) are comparison vs. control values (C, no PAL). *Number signs* (##, ###) are comparison between values obtained for cells grown in the presence of 100 μM PAL and those grown in the presence of 150 μM PAL

To determine the time course and dose-dependency of the inflammatory response induced by elevated PAL levels, we measured ICAM1 protein expression levels in EA.hy926 cells after different exposure times with 100–400 μM PAL (Fig. 1). The results indicate that the inflammatory increases with the duration of exposure and with increasing concentrations of FFA. The most pronounced inflammatory response was found after the 6-day exposure with 400 μM PAL. However, this high-dose, long-term treatment led to an enormous decrease in cell viability that prevented bioenergetic studies. Therefore, to further study the effect of elevated PAL levels on endothelial cells after a chronic 6-day exposure, we used PAL concentrations of 100 and 150 μM. Under these conditions, the ICAM1 expression level was significantly elevated (Figs. 1a and 2a). In addition to the increased ICAM1 expression, treatment with PAL resulted in a dose-dependent decrease in cell viability and density (Fig. 1), indicating the induction of cell death and a reduction of cell proliferation in PAL-treated endothelial EA.hy926 cells.

### In endothelial cells, growth in the presence of high PAL concentrations caused increased fatty acid-supplied respiration and reduced mitochondrial respiration during carbohydrate and glutamine oxidation

**Fig. 3:**
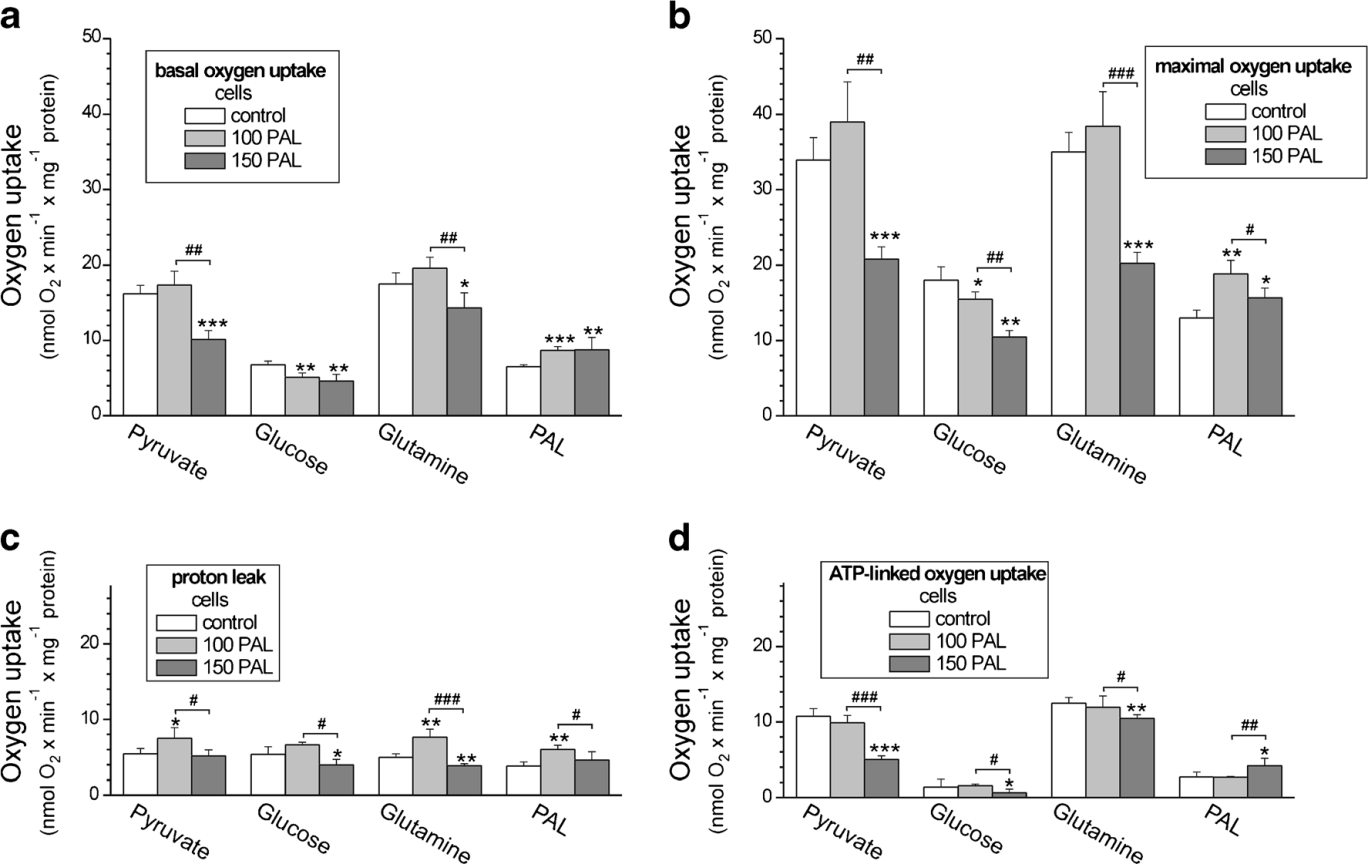
Oxidative metabolism (respiration) of EA.hy926 cells grown for 6 days in the presence of 100 μM PAL (100 PAL), 150 μM PAL (150 PAL), or under control conditions (no PAL). Substrate-dependent changes in the basal oxygen consumption rate (**a**), maximal oxygen uptake (**b**), proton leak (**c**), and ATP-dependent oxygen uptake (**d**). Substrates: 5 mM pyruvate, 5.5 mM glucose, 3 mM L-glutamine, or 0.3 mM PAL. Results are presented as the means ± SD; *n* = 7. *p* < 0.05 (* or #), *p* < 0.01 (** or ##), or *p* < 0.001 (*** or ###). *Asterisks* (*, **, ***) are comparison vs. control values (no PAL). *Number signs* (#, ##, ###) are comparison between values obtained for cells grown in the presence of 100 μM PAL and those grown in the presence of 150 μM PAL

To examine how EA.hy926 cells grown under control or high PAL (100 or 150 μM) conditions respond to simple changes in respiratory substrates, mitochondrial respiratory function (oxygen uptake) was measured. Under basal conditions (basal OCR) (Fig. 3a), FCCP-stimulated conditions (maximal OCR) (Fig. 3b) and in the presence of oligomycin (oligomycin-resistant, ATP-linked OCR) (Fig. 3d), all cells, independent of PAL exposure level during culturing, demonstrated the highest OCR with pyruvate or glutamine and the lowest OCR with PAL as the respiratory substrate. Among the tested OCRs, the non-ATP-linked OCR (proton leak) exhibited the least dependence on the type of reducing substrate present (Fig. 3c).

In general, under all respiratory conditions and with respect to all reducing fuels except PAL, the EA.hy926 cells grown at a higher concentration of PAL (150 μM) displayed a significantly reduced mitochondrial function relative to the cells exposed to control conditions or to 100 μM PAL (Fig. 3). In particular, cells treated with 150 μM PAL exhibited 1.5- to 1.9-fold reductions in their maximal mitochondrial respiratory capacity in the presence of pyruvate, glucose, or glutamine (Fig. 3b). Similarly, with these substrates, a significantly reduced ATP-linked OCR was observed in 150 μM PAL-treated cells, indicating diminished levels of mitochondrial oxidative phosphorylation during carbohydrate and amino acid catabolism (Fig. 3d). Interestingly, in the case of glucose, a decrease in maximal respiratory capacity and basal respiration was found in both types of high PAL-treated cells. For cells grown in the presence of 100 μM PAL, no significant changes in maximal respiration or ATP-linked respiration were observed with pyruvate or glutamine when compared with rates in the control cells. Thus, mitochondrial respiratory measurements indicate a reduction in mitochondrial respiration during carbohydrate and glutamine oxidation in high PAL-treated endothelial cells, especially in those treated with 150 μM PAL.

In contrast, the oxidation of PAL was significantly higher in both of the high PAL-treated cell groups (100 and 150 μM) (Fig. 3b, d). These results indicate that there is a greater contribution from fatty acids as a fuel source for endothelial respiration during growth at high PAL concentrations.

Interestingly, an increase in the non-ATP-linked OCR (proton leak) in the presence of all reducing fuels, including PAL, was observed in cells treated with 100 μM PAL but not in those treated with 150 μM PAL (Fig. 3c). Thus, an elevated uncoupling of respiration was not found in 150 μM PAL-treated cells, whereas a general reduction of aerobic metabolism was observed in these cells, except in the case of FFA oxidation.

### In endothelial cells, growth in the presence of high PAL concentrations did not change mitochondrial biogenesis or aerobic respiration capacity but did reduce fermentation

**Fig. 4:**
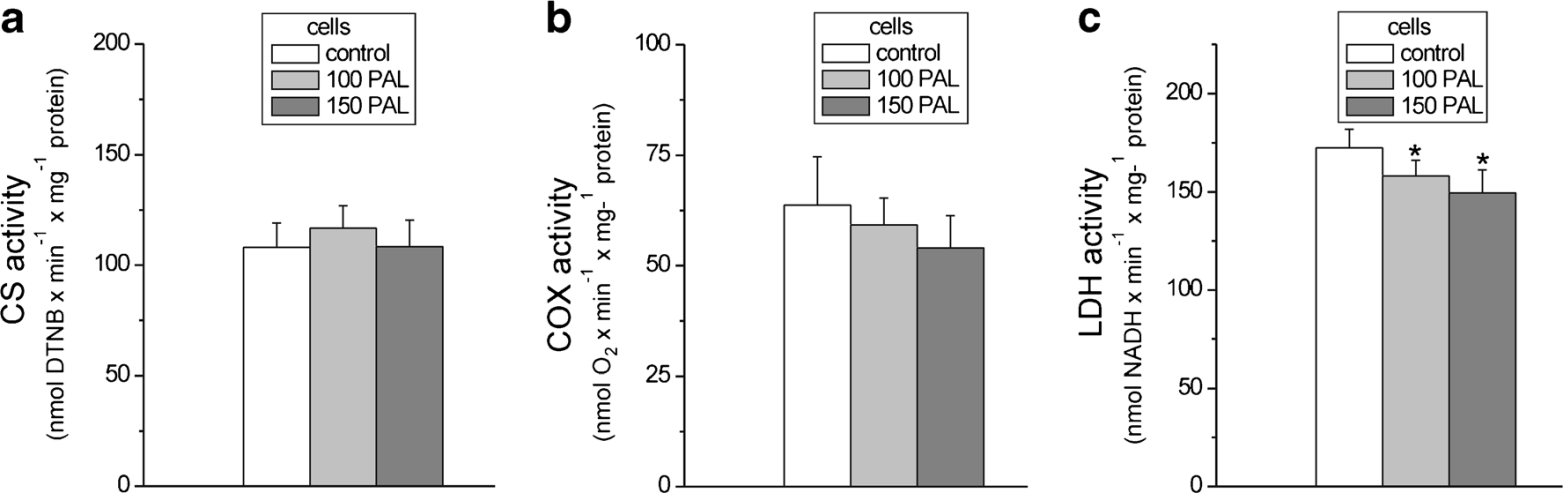
Maximal activities of marker enzymes of aerobic (**a**, **b**) and anaerobic catabolism (**c**) in EA.hy926 cells grown for 6 days in the presence of 100 μM PAL (100 PAL), 150 μM PAL (150 PAL), or under control conditions (no PAL). Enzymes analyzed include citrate synthase (*CS*) (**a**), cytochrome *c* oxidase (*COX*) (**b**), and lactate dehydrogenase (*LDH*) (**c**). Results are presented as the means ± SD; *n* = 7. *p* < 0.05 (*asterisk*), comparison vs. control values (no PAL)

The endothelial cells cultured in the control conditions or high PAL conditions (both at 100 or 150 μM PAL) exhibited similar CS and COX activities (Fig. 4a, b), indicating that the different growth conditions did not change the capacities of the tricarboxylic acid (TCA) cycle or the mitochondrial respiratory chain. Moreover, no differences in the expressions of mitochondrial markers COXII and CS were detected in the control and PAL-treated cells (Fig. 2a). These results indicate that growing in the presence of high PAL concentrations does not change the mitochondrial biogenesis (mitochondrial content) or the cell’s capacity for aerobic respiration.

However, in cells grown at high PAL concentrations, a slight but statistically significant reduction in the activity of LDH, the enzyme that catalyzes the interconversion of pyruvate and lactate, was observed (∼10 and ∼20 % decrease in cells from 100 and 150 μM PAL cultures, respectively) (Fig. 4c). The changes in activity were accompanied by a slight downregulation of LDH at the protein level (Fig. 2a). Moreover, a decrease in the expression of HKI, the enzyme catalyzing the first step of the glycolytic pathway, which is the rate-limiting step, was found in both types of high PAL-treated cells (Fig. 2a). Thus, these results indicate that cells grown at high PAL concentrations display a diminished anaerobic glucose oxidation via the glycolytic pathway and lactic acid fermentation.

### In endothelial cells, growth at high PAL concentrations increased mitochondrial and non-mitochondrial superoxide generation, and the increase was less pronounced in cells treated with 150 μM PAL

**Fig. 5:**
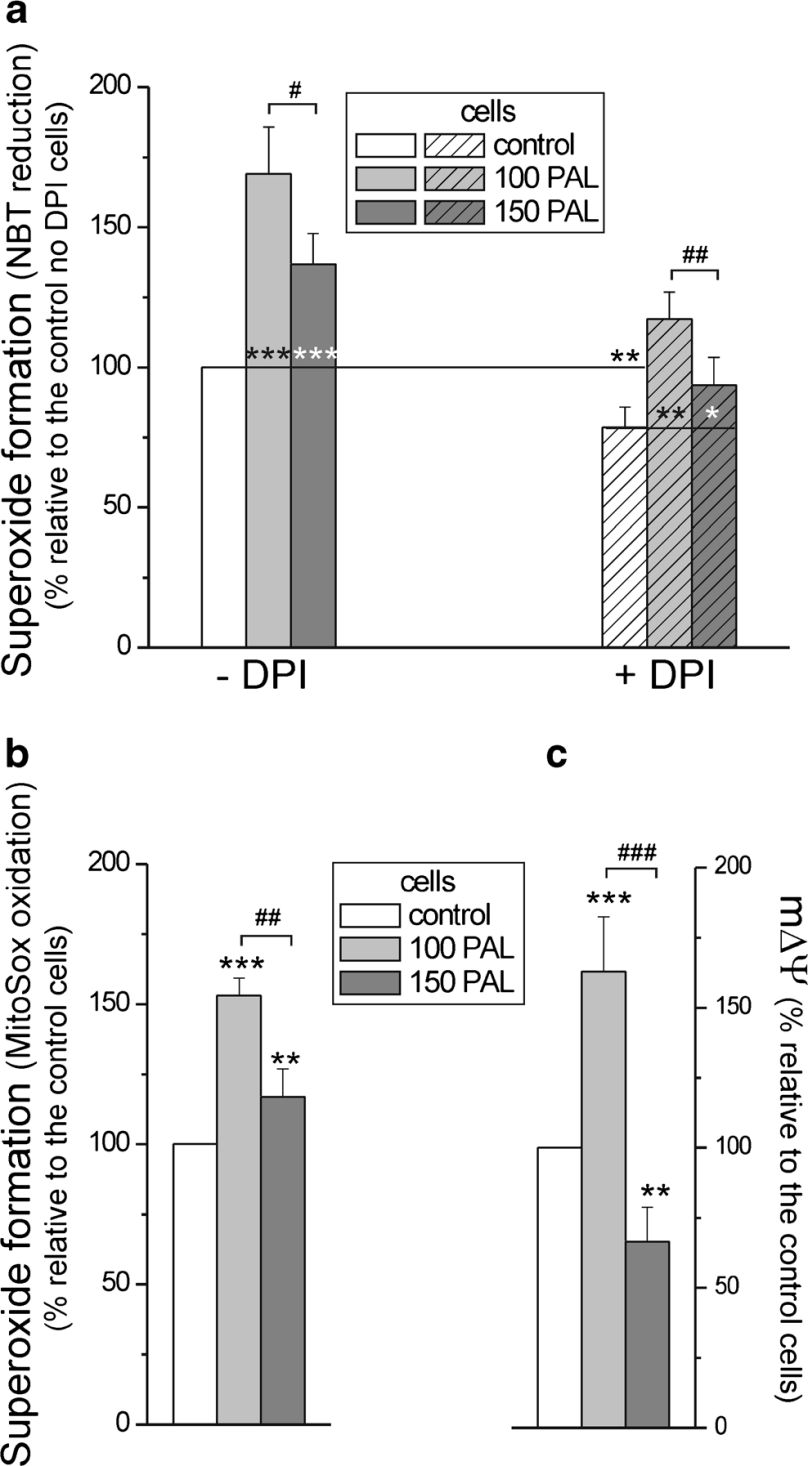
Cellular and mitochondrial superoxide formation (**a**, **b**) and mΔΨ (**c**) in endothelial cells grown for 6 days in the presence of 100 μM PAL (100 PAL), 150 μM PAL (150 PAL), or under control conditions (no PAL). **a** Measurements of superoxide formation with NBT were performed in the absence or presence of 10 μM DPI. **b** Measurements of superoxide formation with the MitoSox probe. Results are presented as the means ± SD; *n* = 8. *p* < 0.05 (*), *p* < 0.01 (** or ##), or *p* < 0.001 (*** or ###). *Asterisks* (*, **, ***) are comparison vs. control values (no PAL). *Number signs* (##, ###) are comparison between values obtained for cells grown in the presence of 100 μM PAL and those grown in the presence of 150 μM PAL

As shown in Fig. 5a, b, compared with the cells cultured under control conditions, the exposure of EA.hy926 cells to high PAL concentrations, both 100 and 150 μM PAL, caused a significant elevation in total and mitochondrial superoxide generation. Mitochondrial superoxide generation was measured either as DPI-insensitive NBT reduction (Fig. 5a) or MitoSOX oxidation (Fig. 5b). A significantly less-pronounced increase in total and mitochondrial superoxide formation was found in cells treated with 150 μM PAL (∼37 and ∼22 % increase, respectively) compared to those treated with 100 μM PAL (∼70 and ∼50 % increase, respectively). We found that in both types of PAL-treated cells, DPI, a flavoprotein inhibitor of NADPH oxidase and eNOS, significantly inhibited (∼30 % reduction) PAL-induced superoxide generation (Fig. 5a). Therefore, in EA.hy926 cells, high PAL-induced ROS production appears to occur via enzyme sources and mitochondrial sources (including DPI-insensitive sources). High PAL-induced mitochondrial and non-mitochondrial ROS production changes were both less pronounced in cells treated with 150 μM PAL than in those treated with 100 μM PAL.

Significant changes in mΔΨ were observed, i.e., ∼60 % increase and ∼30 % decrease, in cells treated with 100 or 150 μM PAL, respectively (Fig. 5c).

### Increased levels of PAL resulting from the growth of cells at high PAL concentrations caused impairment and uncoupling of the mitochondrial oxidative phosphorylation system

**Fig. 6:**
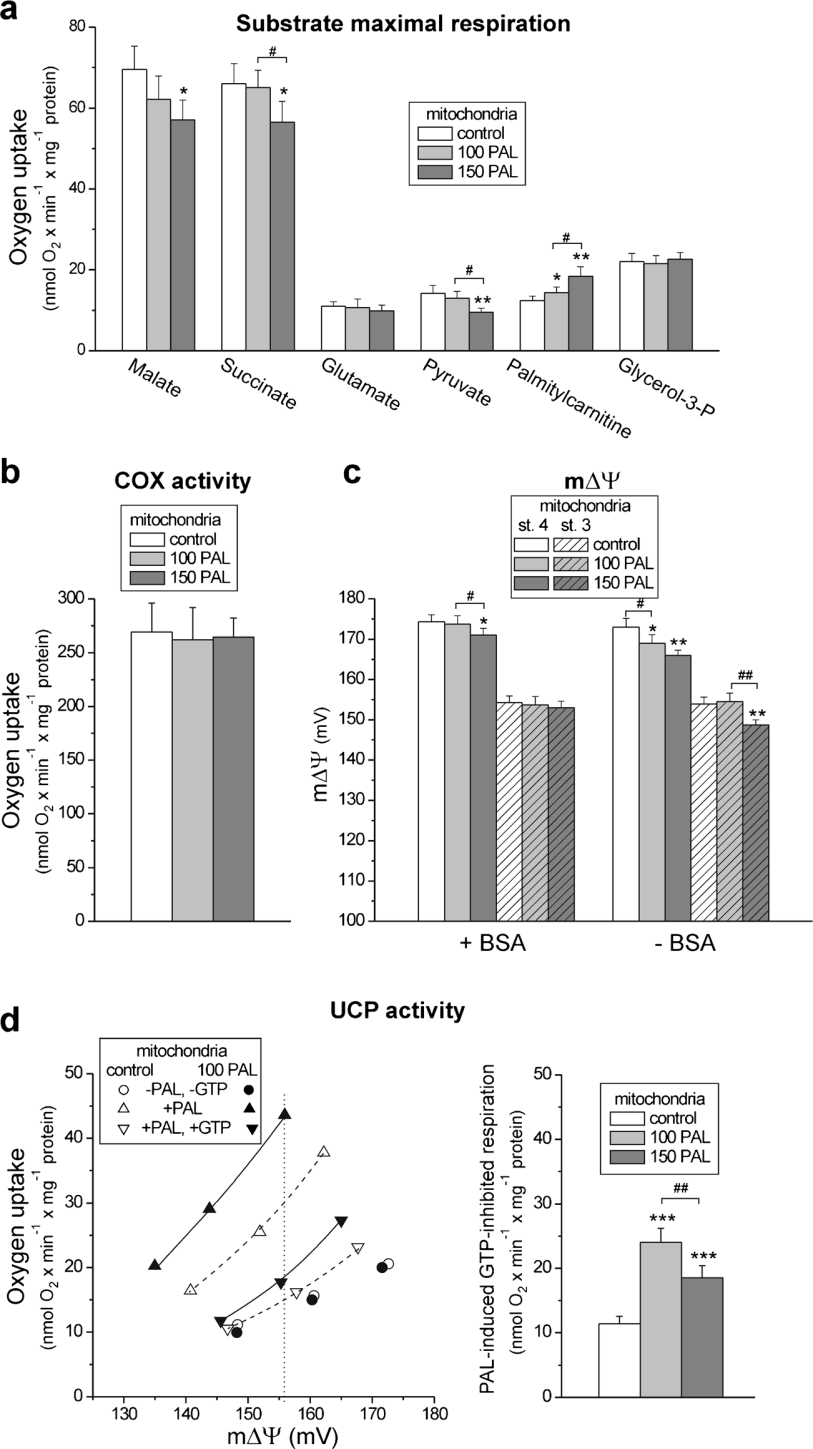
Functional characteristics of endothelial mitochondria isolated from cells grown for 6 days in the presence of 100 μM PAL (100 PAL), 150 μM PAL (150 PAL), or under control conditions (no PAL). **a** The maximal respiration (phosphorylating respiration or uncoupled respiration) in the presence of the following respiratory substrates: 10 mM malate, 10 mM succinate, 10 mM pyruvate, 10 mM glutamate, 0.3 mM palmitoyl*-*DL*-*carnitine, and 3 mM glycerol-3-phosphate (glycerol-3-P). Measurements were performed in the presence of 0.1 % BSA. **b** The COX activity. **c** mΔΨ during the oxidation of malate in the absence of oligomycin and carboxyatractyloside during non-phosphorylating (state 4, *st. 4*) and phosphorylating (state 3, *st. 3*) respiration, with or without 0.1 % BSA. **d** UCP2 activity. Succinate (with 2 μM rotenone) was used as oxidizable substrate. The mitochondria were incubated in the absence or presence of 14 μM PAL and/or 4 mM GTP. Measurements were performed in the presence of 1.8 μM carboxyatractyloside, 0.5 μg/ml oligomycin, and 0.1 % BSA. *Left*: The relationship between the respiratory rate and mΔΨ (proton leak kinetics) during non-phosphorylating succinate oxidation titrated with malonate. Results obtained for mitochondria isolated from cells chronically grown in the presence of 100 μM PAL (100 PAL) are shown. *Right*: The PAL-induced, GTP-inhibited, UCP2-mediated proton leak at the same mΔΨ (156 mV). **a**–**d** Results are presented as the means ± SD; *n* = 4–8. *p* < 0.05 (* or #), *p* < 0.01 (** or ##), or *p* < 0.001 (***). *Asterisks* (*, **, ***) are comparison vs. control mitochondria. *Number signs* (#, ##) are comparison between values obtained for mitochondria isolated from cells grown in the presence of 100 μM PAL and those isolated from cells grown in the presence of 150 μM PAL

To determine the effect of cell growth at high PAL concentrations on respiratory activity at the mitochondrial level, we measured the oxidation of reducing substrates in isolated endothelial mitochondria. Figure 6a shows the maximal mitochondrial respiration rates (phosphorylating or uncoupled respiration) with different respiratory substrates in mitochondria isolated from high PAL-treated and control cells. The highest maximal mitochondrial respiration rate was observed with malate or succinate, substrates which saturate the capacity of the endothelial respiratory chain [14]. Glutamate (an intermediate of amino acid metabolism), glycerol-3-phosphate, and palmitoylcarnitine (lipid breakdown intermediates) were more weakly oxidized by EA.hy926 mitochondria than were malate or succinate (TCA cycle intermediates) (Fig. 6a). Moreover, the oxidation of pyruvate was not as intense as the oxidation of these two TCA cycle intermediates. In mitochondria isolated from cells grown at high PAL concentrations, the oxidation of glutamate and glycerol-3-phosphate remained unchanged. However, the oxidation of pyruvate was significantly reduced by ∼32 % but only in mitochondria from cells treated with 150 μM PAL. This change was accompanied by a downregulation of the E3BP component of pyruvate dehydrogenase (Fig. 2b). Similarly, a higher concentration of PAL in the growth cultures resulted in a decrease in mitochondrial malate and succinate oxidation (Fig. 6a).

Growth under high PAL concentrations did not influence the expression of components of the oxidative phosphorylation system in EA.hy926 mitochondria (Fig. 2b). The expression levels of subunits for ATP synthase (α) and the four respiratory chain complexes, namely, Complex I (NDUFB8), Complex II (subunit 30 kDa), Complex III (Core2), and Complex IV (COXII), were not affected. In addition, no change in the expression level of CS or glutamate dehydrogenase was observed (Fig. 2b). Moreover, the maximal COX activity was also unaffected (Fig. 6b).

The comparison of mitochondrial respiratory activity, coupling parameters (Table 1), and mΔΨ (Fig. 6c) in the absence and presence of 0.1 % BSA, acting as an FFA chelator, indicates that increased levels of PAL resulting from the growth of cells at high PAL concentrations could cause impairment and uncoupling of the mitochondrial oxidative phosphorylation system. In the absence of BSA, (i) a significant decrease in phosphorylating (state 3) respiration, indicating an inhibition of respiratory chain, and (ii) a significant decrease in the ADP/O ratio and respiratory control ratio together with a significant increase in non-phosphorylating respiration, indicating mitochondrial uncoupling, were observed during malate oxidation in mitochondria isolated from cells exposed to high PAL concentrations (Table 1). The highest reduction in phosphorylating respiration and the greatest extent of mitochondrial uncoupling in the absence of BSA were observed in mitochondria from cells treated with 150 μM PAL. In the presence of BSA, these effects were found only for mitochondria isolated from cells treated with 150 μM PAL. Moreover, in non-phosphorylating and phosphorylating malate-oxidizing mitochondria, the mΔΨ was significantly diminished in the absence of BSA, especially in mitochondria from 150 μM PAL-treated cells (by ∼7 and ∼5 mV, respectively) (Fig. 6c). Changes in mΔΨ (Fig. 6c) reflect changes in respiratory rates (Table 1). In the absence of BSA, in non-phosphorylating mitochondria, the decrease in mΔΨ (Fig. 6c) was accompanied by an increase in respiratory rate (Table 1) indicating increased mitochondrial uncoupling. In phosphorylating mitochondria, the decrease in mΔΨ (Fig. 6c) was accompanied by a decrease in respiratory rate (Table 1), indicating inhibition of mitochondrial respiratory chain. In the presence of BSA, simultaneous changes in mΔΨ (a decrease) and respiratory rate (an increase) were only observed for non-phosphorylating mitochondria from cells grown at the higher PAL concentration. It seems that 0.1 % BSA was able to chelate excess FFAs only in mitochondria from cells treated with 100 μM PAL. Moreover, mitochondria isolated from cells treated with 150 μM PAL exhibited a slight but statistically significant reduction in the integrity of the outer mitochondrial membrane (96.04 ± 0.39 %, SD, *n* = 8) compared to mitochondria from control cells (98.01 ± 0.69 %, SD, *n* = 8) and those treated with 100 μM PAL (97.11 ± 0.63 %, SD, *n* = 8). The above results indicate that increased levels of PAL resulting from the growth of cells at high PAL concentrations could cause impairment and uncoupling of the oxidative phosphorylation system in endothelial mitochondria.

**Table 1:** Respiratory rates and coupling parameters in endothelial mitochondria isolated from cells grown for 6 days in the presence of 100 μM PAL (100 PAL) or 150 μM PAL (150 PAL)

Mitochondria			
	Control	100 PAL	150 PAL
Malate + BSA			
State 4	19.2 ± 2.0	19.3 ± 2.2	25.0 ± 2.4*, #
State 3	71.0 ± 0.6	65.0 ± 0.6	62.5 ± 0.5*
RCR	3.7 ± 0.3	3.6 ± 0.3	2.5 ± 0.3**, ##
ADP/O	2.25 ± 0.20	2.21 ± 0.21	2.20 ± 0.18
Malate − BSA			
State 4	21.3 ± 2.0	24.3 ± 2.2*	29.0 ± 2.4***, ##
State 3	72.3 ± 0.5	65.0 ± 0.6	59.5 ± 0.5**, #
RCR	3.4 ± 0.2	2.7 ± 0.3*	2.1 ± 0.1***, ##
ADP/O	2.20 ± 0.22	2.07 ± 0.16*	1.82 ± 0.18**, #

Respiratory rates were measured in the absence (state 4, non-phosphorylating respiration) or presence (state 3, phosphorylating respiration) of 150 μM ADP with 10 mM malate. The respiratory rates (in nmol O_2_ × min^−1^ × mg^−1^ protein), and respiratory control ratios (RCR) and ADP/O ratios are presented. Results are presented as the means ± SD; *n* = 6. *Asterisks* mean comparison vs. control mitochondria. *Number* *signs* mean comparison between values obtained for mitochondria from cells grown in the presence of 100 μM PAL and those from cells grown in the presence of 150 μM PAL

*p < 0.05; **p < 0.01; ***p < 0.001; #*p* < 0.05; ##*p* < 0.01

### Growth under high PAL conditions induced an increase in the mitochondrial oxidation of palmitoylcarnitine

In contrast to the oxidation of other respiratory substrates, the mitochondrial oxidation of palmitoylcarnitine was significantly increased by ∼16 and ∼48 % in mitochondria isolated from endothelial cells treated with 100 and 150 μM PAL, respectively (Fig. 6a). Moreover, in EA.hy926 mitochondria, a high PAL level during cell growth increased (by ∼70 and ∼130 % for mitochondria from cells treated with 100 and 150 μM PAL, respectively) the expression of acyl-CoA dehydrogenase (ACADS) (Fig. 2b), which catalyzes the initial step of fatty acid β-oxidation. These findings are consistent with the greater oxidation of PAL that was observed in both types of the high PAL-treated cells (Fig. 3), indicating a greater oxidation of reducing substrates originating from fatty acid β-oxidation.

### Growth under high PAL conditions induced increased UCP2 activity

Using a Western blot analysis, the expression of UCP2 and UCP3, but not UCP1, could be detected in EA.hy926 cells [26]. In our study, the analysis of UCP2 protein expression (Fig. 2b) showed a considerable upregulation of the protein in mitochondria isolated from high PAL-treated cells relative to those isolated from untreated control cells. Namely, ∼2.5-fold and ∼2-fold elevations in UCP2 protein levels were found in mitochondria from cells treated with 100 and 150 μM PAL, respectively, relative to the level in the control cells. However, endothelial cells grown at high PAL concentrations exhibited UCP3 expression levels in the mitochondria that were similar to that of the control mitochondria (Fig. 2b). Because it seems that UCP2 is the only UCP involved in the high-PAL-induced modifications that occur in EA.hy926 endothelial cells, we attribute the high PAL-induced changes in UCP activity, described below, to UCP2.

To determine whether UCP2 activity is changed due to the growth of EA.hy926 cells with high PAL concentrations, we evaluated the activation of UCP2 by FFAs (PAL) and the inhibition by GTP in isolated endothelial mitochondria (Fig. 6d). In general, in non-phosphorylating mitochondria isolated from cells grown under high PAL conditions, the stimulatory effect of PAL and the inhibitory effect of GTP were considerably stronger than the effects observed in the control mitochondria. Figure 6d (*left panel*) shows an example of the relationship between the oxygen consumption rate and the mΔΨ (flux-force relationship) during succinate oxidation titrated with malonate for mitochondria isolated from cells treated with 100 μM PAL. An analysis of the proton leak kinetics indicates that for specific PAL (14 μM) and GTP (2 mM) concentrations, the PAL-induced, GTP-inhibited, UCP2-mediated proton leak (UCP2 activity) at the same mΔΨ (156 mV) was ∼2-fold and 1.6-fold higher in mitochondria from the cells treated with 100 and 150 μM PAL, respectively, than it was in the control mitochondria (Fig. 6d, *right panel*).

Thus, these results indicate that in response to high PAL concentrations during endothelial cell growth, UCP2 activity and protein levels are considerably increased, but the increase is less pronounced in mitochondria from the cells grown at a higher PAL concentration (150 μM PAL). Similarly, superoxide dismutase 2 (SOD2), another mitochondrial antioxidant protein was upregulated in both types of high PAL-treated cells (Fig. 2b).

## Discussion

Our results show that the treatment of endothelial cells with high PAL levels resulted in an increase in cell inflammatory response and a marked decrease in cell viability and density, indicating the induction of cell death and a reduction of cell proliferation in PAL-treated EA.hy926 cells. The lipotoxic effects of high levels of FFAs on endothelial cells have been described previously. Human brain microvascular endothelial cells (HBMVECs) exposed to FFAs show significantly decreased cell proliferation, increased apoptosis and intracellular ROS formation, and decreased mΔΨ [31]. In human coronary artery endothelial cells (hCAEC), exposure to 100 μM PAL for 24 h has been shown to induce a greater than 2-fold increase in apoptosis [2]. Direct apoptotic effects of FFAs suggest that inappropriate elevations in FFAs may affect vascular endothelium by impairing cell survival via the activation of apoptosis, thus contributing to the development of cardiovascular disease in type 2 diabetic patients [19]. Interestingly, in endothelial EA.hy926 cells, exposure to pathological PAL concentrations has been shown to induce a lipotoxic response that finally leads to Ca^2+^-dependent autophagy and necrotic cell death [12]. It must be emphasized that in our study, in which we handle endothelial cell populations that are differently treated with potentially (at high concentrations) cytotoxic PAL, individual experimental readouts might be affected by dead cells. The results obtained with cells should be therefore interpreted with caution.

The aim of our study was to determine the effects of high FFA levels on mitochondrial oxidative metabolism in endothelial cells, including the effects on isolated endothelial mitochondria. First, we determined how aerobic metabolism in endothelial EA.hy926 cells supplied with different reducing fuels was altered by long-term cell growth in the presence of high PAL concentrations. The comparison of the mitochondrial respiratory functions of EA.hy926 cells cultured in the medium with either high PAL levels or no PAL demonstrate that chronic high PAL conditions, especially treatment with 150 μM PAL, induce a reduction in mitochondrial respiration supplied with carbohydrate catabolic intermediates (glucose or pyruvate) and glutamine, whereas cellular respiration with PAL (an intermediate in lipid metabolism) was considerably increased. Moreover, in EA.hy926 mitochondria from cells grown at high PAL levels, the mitochondrial oxidation of palmitoylcarnitine and the expression of acyl-CoA dehydrogenase were significantly increased. However, in mitochondria from cells treated with 150 μM PAL, the oxidation of pyruvate and the expression of the E3BP component of pyruvate dehydrogenase were significantly reduced. Thus, our results show for the first time that high-PAL conditions lower the contribution of carbohydrate and amino acid oxidation to the aerobic metabolism of endothelial cells, whereas the contribution of FFA oxidation increases. Interestingly, it has been shown previously that in human umbilical vein endothelial cells (HUVECs), fatty acids can serve as an important energy source; moreover, the activation of AMP-activated protein kinase (AMPK), a major factor in modulating the response of the endothelium to stresses that alter its energy state, favors the oxidation of FFAs as the source of ATP production [4].

In EA.hy926 cells grown for 6 days at high PAL concentrations, the decreased respiration in the presence of glucose was accompanied by a reduced expression of hexokinase I and a decrease in LDH activity. Thus, in addition to a reduction in aerobic glucose oxidation, endothelial cells grown at high PAL concentrations seem to also display a diminished anaerobic glycolysis. To the best of our knowledge, this is the first observation of the effect of high PAL levels on glucose catabolism in endothelial cells.

It has been well documented that the exposure of endothelial cells to high FFA levels leads to increased intracellular ROS production and therefore excessive oxidative stress [3, 6, 8, 31]. Under our experimental conditions, the increased oxidative stress in EA.hy926 cells grown under chronic high PAL conditions was demonstrated by a significant increase in intracellular and mitochondrial ROS generation and an upregulation of the expression of proteins of the mitochondrial antioxidative system, such as SOD2 and UCP2. However, high PAL-induced mitochondrial ROS production was less pronounced in cells treated with 150 μM PAL than in those treated with 100 μM PAL, which was likely due to the considerable reduction in cell viability in these cells. It seems that at excessively high PAL levels (150 μM in our study), endothelial cells are no longer able to protect against the overwhelming oxidative stress, and their antioxidant system is no longer sufficient.

A surprising reversal of observed effects (i.e., a less pronounced increase or even decrease in observed changes) when comparing 100 and 150 μM PAL-treated cells to control cells was found in the (i) expression of mitochondrial antioxidant proteins UCP2 (and activity) and SOD2, (ii) mitochondrial ROS formation, and (iii) mΔΨ. Taking all into consideration, it appears that differences in these parameters are due to differences in cell death between 100 and 150 μM PAL-treated endothelial cells. In 100 μM PAL-treated cells, the increase in mitochondrial membrane potential, ROS formation, and expression of antioxidant proteins indicates an important contribution of apoptotic death. In contrast, in 150 μM PAL-treated cells, a less pronounced increase in mitochondrial ROS formation and expression of antioxidant proteins as well as a decrease in mΔΨ indicate an increased contribution of necrotic death. This finding is in line with a recent report, in which PAL-induced necroptosis of endothelial EA.hy926 cells has been described [12].

Mitochondria are not only a major intracellular source of ROS but are also a sensitive target for oxygen radicals. Mitochondria contribute to cell death by reducing ATP production, increasing ROS production, and releasing apoptotic regulatory and signaling molecules from the intermembrane space [17]. Therefore, the high PAL-induced lipotoxicity reflected by the marked decrease in cell viability of cultured endothelial EA.hy926 cells could result from elevated mitochondrial ROS generation and disturbances of mitochondrial aerobic metabolism. Interestingly, in obese Zucker rats and high fat-diet-induced obese mice, the inhibition of FFA release from adipose tissue and the inhibition of the rate-limiting enzyme in mitochondrial FFA oxidation reduces aortic ROS production and prevents prostacyclin synthase and eNOS inactivation [6].

Because the activities of COX and CS and the expression level of mitochondrial marker proteins remained unchanged, it appears that the growth of EA.hy926 cells at high PAL concentrations did not change their maximal aerobic respiration capacity or mitochondrial biogenesis. However, measurements of mitochondrial function in isolated mitochondria indicate that increased levels of PAL resulting from exposure to high PAL concentrations do not only cause uncoupling but also impair oxidative phosphorylation in endothelial mitochondria. Thus, in addition to the findings from studies using animal models and those on human subjects demonstrating the detrimental effects of FFAs on vascular health [30], our study shows for the first time that the chronic exposure of cultured endothelial cells to high levels of FFAs leads to mitochondrial oxidative dysfunction. The inhibition of mitochondrial respiration and the decrease in ATP synthesis yield by excessive FFA levels could be an important factor of endothelial lipotoxicity.

Our results indicate that, in response to high PAL concentrations during endothelial cell growth, UCP2 activity and protein levels are considerably increased in mitochondria. The increase was less pronounced in mitochondria from the cells grown at a higher PAL concentration (150 μM PAL), where the excess of FFAs likely led to the impairment of the oxidative phosphorylation system, thereby decreasing the mΔΨ, which is the driving force of UCPs. We have shown previously that in endothelial EA.hy926 cells, the high glucose-induced increase in the activity of UCP2 reduces mitochondrial ROS formation and improves cellular resistance to stress [13], although a greater reduction in the yield of oxidative phosphorylation is observed [14]. Thus, one physiological role of UCP2 could be the attenuation of mitochondrial ROS production under conditions of oxidative stress, particularly given that endothelial mitochondria are not particularly dependent on oxidative phosphorylation [14]. UCPs are known to be upregulated in some mammalian tissues when FFA availability exceeds mitochondrial oxidative capacity [11]. Our results confirm previous implications that UCP2 may serve as a sensor and a negative regulator of mitochondrial ROS production in endothelial cells with elevated FFAs levels, protecting mitochondria from FFA-induced damage. Beyond that, UCP2/3 have been suggested to take part in the export of the fatty acid anions that can accumulate in the mitochondrial matrix during mammalian cell overload [22] or in the process of removing the lipid peroxides from the matrix [9]. The knockdown of UCP2 in mice fed a high-fat diet has been shown to cause a reduction in antioxidant capacity, endothelial dysfunction, and atherosclerotic deterioration [18].

**Fig. 7:**
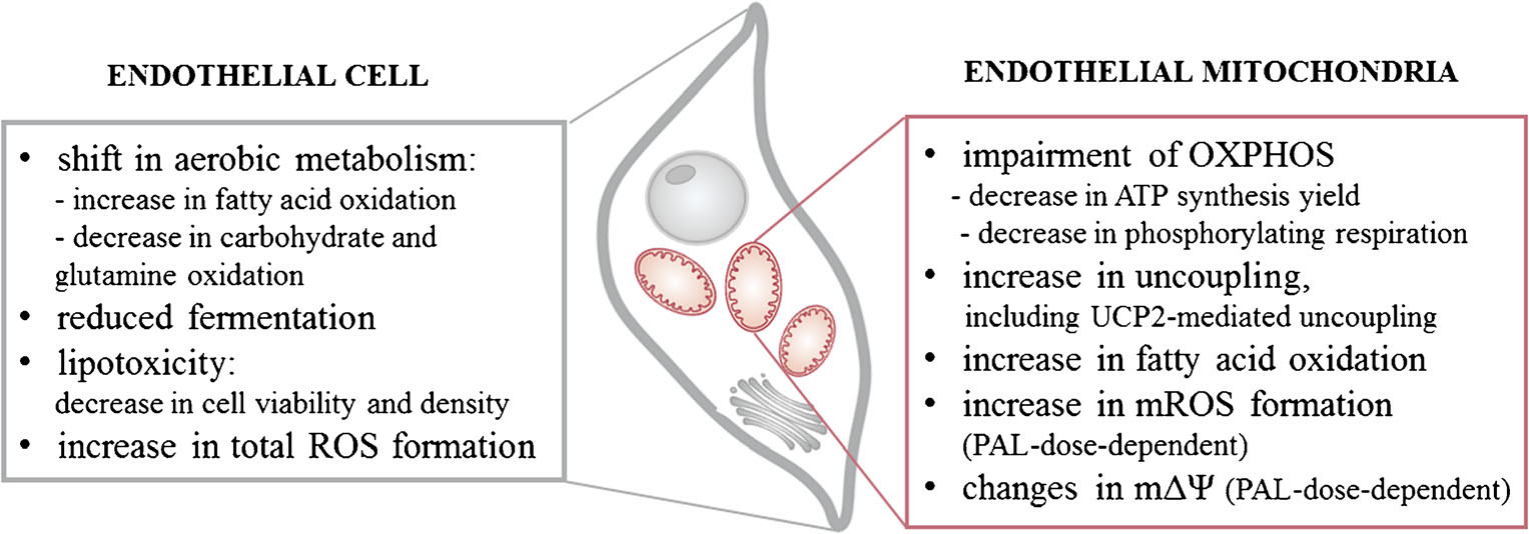
Effects of chronic exposure to high PAL concentrations on endothelial cells and their mitochondria, which are observed in this study. *OXPHOS* oxidative phosphorylation

In conclusion, the growth of endothelial cells under high PAL conditions induces numerous changes in their aerobic metabolism (Fig. 7), particularly a shift toward the intensified oxidation of FFAs. High levels of FFAs induce increased intracellular and mitochondrial ROS production and therefore excessive oxidative stress, which damages endothelial cells and results in decreased cell viability. Observed differences in mitochondrial ROS formation, mΔΨ, and expression of mitochondrial antioxidant proteins, when comparing 100 and 150 μM PAL-treated cells, may be due to differences in the PAL-induced cell death pathways (dominance of apoptosis or necroptosis, respectively). However, further studies are necessary to elucidate this phenomenon. Our results show also that excess FFAs could result not only in elevated mitochondrial uncoupling, including UCP2-mediated uncoupling, but also in impaired mitochondrial function. These observations highlight the role of endothelial mitochondria in response to metabolic disturbances related to high levels of FFAs.
